# Beyond the Bedside: How Moral Sensitivity and Self-Efficacy Shape Person-Centered Care in Nursing

**DOI:** 10.1155/jonm/2823773

**Published:** 2025-09-25

**Authors:** Noushin Mousazadeh, Maysam Rezapour, Faezeh Babaieasl, Hamid Sharif-Nia, Sogand Mivehchi

**Affiliations:** ^1^Department of Nursing, Amol School of Nursing and Midwifery, Mazandaran University of Medical Sciences, Sari, Iran; ^2^Department of Paramedicine, Amol School of Paramedical Sciences, Mazandaran University of Medical Sciences, Sari, Iran; ^3^Department of Nursing Practice, College of Nursing and Health Sciences, Texas A&M University, Corpus Christi, Texas, USA; ^4^Psychosomatic Research Center, Mazandaran University of Medical Sciences, Sari, Iran; ^5^Research Committee of Mazandaran University of Medical Sciences, Sari, Iran

**Keywords:** moral sensitivity, nurses, patient-centered care, person-centered care, self-efficacy

## Abstract

**Background:** Person-centered care (PCC) enhances the quality of care, safety, and satisfaction, yet many patients still do not consistently receive it. Evidence suggests that nurses' self-efficacy and moral sensitivity are necessary for implementing PCC, but both remain understudied, and many nurses show low levels of moral sensitivity. Few studies, particularly in Iran, have investigated these factors within multilevel contexts. This study examines this gap by exploring the relationship between moral sensitivity, self-efficacy, and PCC among hospital nurses in northern Iran.

**Methods:** This cross-sectional study was conducted between 2022 and 2023 with 247 nurses from four public hospitals in northern Iran. Data were collected using validated Persian versions of the Person-Centered Care Assessment Tool, Moral Sensitivity Questionnaire, and General Self-Efficacy Scale. To account for the nested structure of the data (nurses within wards), a two-level hierarchical linear model was employed. This analysis assessed the associations of individual and ward-level collective moral sensitivity and self-efficacy with PCC, while adjusting for key demographic variables.

**Results:** Two-level linear regression models were employed to examine the associations between moral sensitivity, general self-efficacy, and person-centered nursing, while adjusting for demographic variables and collective scores of moral sensitivity and general self-efficacy at the ward level. The analysis revealed that individual nurses' (Level 1) perceptions of moral sensitivity demonstrated a significant positive association with person-centered nursing (*B* = 0.271, *p* < 0.001). Also, the majority of participants had over 5 years of work experience (46.6%) and most of them had a monthly income between 6 and 12 million Tomans (51.1%). The intraclass correlations (ICC) were calculated for all four models. The empty model indicated that 13.21% of the variance in PCC was attributed to ward level factors. However, in model 4, following the inclusion of contextual variables at the ward level, the ICCs decreased to 4.84%. This reduction suggests that a portion of the variance was explained by the collective moral sensitivity and general self-efficacy variables at the ward level.

**Conclusion:** The moderate level of PCC observed among nurses may indicate the need for implementing strategies to enhance their comprehension of this concept. Given the critical role of moral sensitivity in delivering PCC, it is advisable to prioritize the recruitment of nurses with robust ethical competencies. Additionally, providing in-service learning opportunities and organizational support is recommended to cultivate moral sensitivity and elevated care quality.

## 1. Introduction

Dr. Jean Watson's conceptualization of caring behavior laid a foundational cornerstone for contemporary nursing practice. Person-centered care (PCC) has emerged as a prominent nursing care model [1, 2], distinguished by its emphasis on patients' active involvement in their treatment regimens [3] and the recognition of their agency in care-related decision-making [4]. Given PCC's established importance to quality healthcare delivery and patient safety, its provision is associated with improvements in the work environment, reduced operational costs, and enhanced patient and nurse satisfaction [3–5]. Consequently, the effective implementation of PCC is crucial for optimizing care quality and enhancing organizational efficiency within healthcare settings [6]. However, despite these recognized benefits, a substantial proportion, approximately 40%, of hospitalized patients do not consistently receive PCC [7]. This gap underscores the imperative for healthcare providers to cultivate a comprehensive understanding of patients' needs and the multifaceted factors that influence the consistent delivery of PCC [8, 9].

Despite the well-substantiated advantages of PCC and the pivotal role of nurses in its effective implementation, a discernible gap persists in our understanding of the translational processes required to shift from traditional, provider-centric care models to a PCC-oriented approach. Addressing this knowledge deficit is therefore of paramount importance to delineate the key factors influencing PCC delivery [10, 11]. Prior investigations suggest that an array of relational variables, including empathy, moral sensitivity, prosocial behaviors, and interpersonal respect, exert a significant influence on the provision of PCC within clinical settings [12–14].

Self-efficacy, a cornerstone concept in Bandura's social cognitive theory, denotes an individual's intrinsic belief in their capacity to execute specific tasks effectively [15]. This construct exerts a profound influence on nurse–patient interactions, thereby directly impacting the quality of patient care delivery [16, 17]. Individuals exhibiting higher levels of self-efficacy demonstrate a greater propensity for innovation, active task engagement, and goal-oriented perseverance. Empirical evidence suggests that self-efficacy serves as a crucial bridge between theoretical knowledge and practical application, enabling nurses with elevated self-efficacy to provide superior care. Conversely, those with diminished self-efficacy often exhibit task avoidance behaviors and reduced performance efficacy [18–20]. Recent research from 2021 has elucidated the significant impact of both self-efficacy and empathy on PCC delivery [21]. However, despite its pivotal role in enhancing care quality and fostering nurses' professional success across diverse career domains, the body of research examining self-efficacy within the nursing context remains notably limited [22].

Moral sensitivity constitutes another salient construct influencing the delivery of PCC. It assumes a pivotal role in nurses' ethical decision-making processes [23], encompassing their acute awareness of core ethical values and the potential ramifications of their actions on patient well-being [19]. As integral members of the interprofessional healthcare team, nurses must cultivate a heightened attunement to the ethical dimensions inherent in their practice to uphold patients' rights in autonomous decision-making and to navigate complex ethical challenges effectively [13, 23, 24]. Given their demanding workloads and frequent interactions with vulnerable individuals, nurses routinely encounter ethical dilemmas within their professional environments and bear the responsibility of providing care that adheres rigorously to established ethical standards [24, 25]. Accordingly, moral sensitivity is indispensable for ensuring the provision of truly PCC [12]. However, despite its recognized importance, recent empirical evidence suggests that a substantial proportion approximately 55.5% of nurses exhibit comparatively low levels of moral sensitivity [25].

A limited number of studies have explored the correlation of PCC among hospital nurses. Also, studies demonstrated moderate moral sensitivity with regional variability, different nurse perceptions of PCC, and an important role of moral sensitivity in predicting the quality of care. However, the relationship between moral sensitivity, self-efficacy, and PCC remains underexplored, and organizational and environmental factors may influence PCC delivery [26–29]. This multilevel approach facilitates the examination of personal attributes, ward-specific characteristics, and contextual influences on care delivery. Variables such as sex, age, employment status, educational attainment, and marital status are frequently investigated as potential correlates of PCC, while psychological constructs like resilience can be assessed at both individual and collective levels of analysis [30, 31]. Given the influential role of hospital wards in shaping nurse behavior, ward-level factors, such as shared perceptions of resilience, have also been linked to PCC provision [32].

Empirical investigations of PCC among hospital nurses in Iran, particularly those accounting for both individual and ward-level variations, remain scarce. Existing research tends to prioritize individual-level resilience perceptions, often neglecting the potential influence of collective resilience dynamics at the ward level. Therefore, this study aims to address this gap by examining the association between moral sensitivity, general self-efficacy, and PCC among hospital nurses in northern Iran, utilizing multilevel contextual effects modeling to account for the nested structure of the data.

## 2. Methods

### 2.1. Design

This cross-sectional study was conducted between December 2022 and February 2023. A convenience sampling method was employed to recruit 247 nurses working in four general hospitals located in northern Iran. This design was chosen for its feasibility in capturing a snapshot of these variables at a single point in time. However, the authors acknowledge that this approach limits the ability to infer causality or assess changes over time. Additionally, the convenience sampling and regional focus may limit the generalizability of the findings to the broader nursing population.

## 3. Participants and Sampling

The study participants were selected based on the following criteria. Inclusion required being a full-time registered nurse in a general hospital, holding a minimum of a bachelor's degree in nursing, possessing at least 12 months of clinical experience, and being actively employed in a bedside nursing role. Conversely, nurses working part-time or in nonclinical capacities, those with less than 12 months of clinical experience, individuals not directly engaged in bedside patient care, and those who declined to provide informed consent were excluded from the study.

### 3.1. Dependent Variable

PCC, operationalized through latent profile analysis (LPA), served as the dependent variable. LPA identified unobserved subgroups based on responses to the 13-item Person-Centered Care Assessment Tool (P-CAT). Profile determination utilized statistical criteria and theoretical interpretability.

### 3.2. Control Variables

The demographic variables included age (in years), sex (male/female), employment status (full-time/part-time), education level (bachelor's/master's & PhD), marital status (married/single/divorced/widowed), and the ward in which the nurse was currently working (categorized by ward type: Medical, Surgical, ICU, etc.). These variables were included to control for potential confounding effects on the relationship between moral sensitivity, self-efficacy, and PCC, as prior research has demonstrated their associations with nurses' attitudes, behaviors, and patient care outcomes.

### 3.3. Independent Variables

The study utilized moral sensitivity and general self-efficacy as independent variables. These constructs were measured using the moral sensitivity and the general self-efficacy, respectively, as described in detail below.

### 3.4. Data Collection

Data were collected via a self-administered questionnaire distributed to participants within their respective hospital wards. Prior to questionnaire completion, all participants received a detailed informed consent form and were instructed to review it thoroughly. Following consent, participants anonymously completed the questionnaire, with an average completion time of 20–25 min. To uphold data integrity, all returned questionnaires were scrutinized for missing entries or inconsistencies. When necessary, participants were contacted directly to resolve any ambiguities or incomplete responses.

### 3.5. Instruments

The questionnaire comprised four sections: a Sociodemographic Characteristics Form, P-CAT, the Moral Sensitivity Questionnaire (MSQ), and the General Self-Efficacy Scale (GSE). The questionnaires were originally in English but were translated into Persian (Farsi) following a standard forward–backward translation protocol to ensure conceptual equivalence.

#### 3.5.1. Self-Designed Sociodemographic Characteristics Form

This form, developed following a preliminary review of the literature, included items assessing nurses' age, sex, years of work experience, marital status, education level, and type of shift. The form was pretested on a small sample (*n* = 5) of nurses to assess its clarity and comprehensiveness.

#### 3.5.2. P-CAT

The P-CAT, developed and validated by Edvardsson et al. in Australia in 2010, measures the extent to which staff experience the care environment as person-centered [33]. The psychometric properties of this questionnaire have been examined in several studies [34, 35]. Cronbach's alpha coefficient in a Chinese study by Li et al. ranged from 0.91 to 0.94, indicating strong internal consistency. The P-CAT comprises 13 items assessing three dimensions: personalized care, organizational support, and environmental accessibility. Responses are rated on a five-point Likert scale ranging from “*I completely disagree*” [1] to “*I completely agree*” [5], with total scores ranging from 13 to 65. Higher scores indicate greater PCC [33]. In the present study, Cronbach's alpha for the P-CAT was 0.73.

#### 3.5.3. MSQ

The MSQ, developed by Lützen et al. in Sweden, consists of 25 items measuring nurses' moral sensitivity in clinical practice [36]. Each item is rated on a five-point scale: completely agree [4], agree [3], disagree [2], completely disagree [1], and neither agree nor disagree (0). While the original scoring guide suggests scores from 0 to 50 indicate low moral sensitivity, 51–75 moderate, and 76–100 high, this is incorrect given the scoring system with the “neither agree nor disagree option”. With this system, an overall score is calculated, but there are not set ranges for high/med/low sensitivity. The MSQ also assesses six dimensions of moral sensitivity: (1) respect for patient autonomy (items 10, 12, 13), (2) awareness of communication (items 1, 2, 3, 4, 17), (3) professional knowledge (items 16, 24), (4) experience of ethical problems and conflicts (items 9, 11, 15), (5) application of ethical concepts in ethical decision-making (items 6, 8, 14, 18, 20), and (6) honesty and benevolence (items 5, 7, 19, 21, 23, 25) [35]. In the present study, Cronbach's alpha was 0.78.

#### 3.5.4. GSE

The GSE, designed and validated by Scherer, Maddox, and colleagues in 1982, comprises 17 items assessing three dimensions: willingness to initiate, willingness to exert effort, and resistance to obstacles [37]. Items are rated on a five-point Likert scale ranging from “*completely disagree*” [1] to “*completely agree*” [5]. Total scores range from 17 to 85. While a common interpretation suggests score ranges for low/average/high self-efficacy levels, there are no set cut-offs for each. Law and colleagues confirmed the validity and reliability of this scale in Sweden in 2011, reporting a Cronbach's alpha of 0.90 [38]. In the present study, the internal consistency was 0.85.

### 3.6. Data Analysis

Descriptive statistics were employed to describe the sample characteristics and the distributions of the dependent and independent variables, including means, standard deviations, ranges, and percentages. Factor scores were obtained from the sum of items in each scale used in subsequent regression analyses. Since nurses are nested within their wards, hierarchical generalized linear models (HGLM) were considered as a possible analytical method to address nonindependence and clustering. Four models were run: initially, an empty model with no predictors (Model 1) was executed, followed by the addition of individual-level characteristics (age, sex, marital status, salary, and experience in the current ward) (Model 2), then moral sensitivity and general self-efficacy (Model 3), and finally, the collective scores of moral sensitivity and general self-efficacy at the ward level (Model 4). Additionally, the variance components (random effects) of “person-centered nursing” were estimated in all models to account for ward-level variance. Analyses were conducted using STATA Version 17, employing the restricted (or residual) maximum likelihood (REML) estimation method to obtain unbiased estimates of variance component parameters. The Level 1 model can be represented as

(1) $$\begin{array}{c} Y_{ij}=\beta_{0j}+\beta_{1j}*\text{age}+\beta_{2j}*\text{gender}+\beta_{3j}*\text{marital status}+\beta_{4j}*\text{salary}+\beta_{5j}*\text{out}–\text{experience in current ward}{\text{⁣}2–5\text{ years}+\beta_{6j}*\text{out}–\text{experience in current ward}>5\text{ years}+\beta }_{7j}*\text{moral sensitivity}+\beta_{8j}*\text{general self}‐\text{efficacy }{+r}_{ij}, \end{array}$$

where *Y*_*ij*_ is the outcome variable (person-centered nursing); *β*_0*j*_ is the intercept; *β*_1*j*_, *β*_7*j*_, and *β*_8*j*_ are the linear relationships of the age, moral sensitivity, and general self-efficacy with the person-centered nursing, respectively; *β*_2*j*_, *β*_3*j*_, and *β*_4*j*_ are gender difference the difference in sex, marital status, and salary category, respectively; and *β*_5*j*_ and *β*_6*j*_ are coded to represent the difference in the out-experience in current ward where the < 2 years was the reference group. The Level 2 model modeled the collective scores of moral sensitivity and general self-efficacy at the level. The Level 2 model is represented below predicting the ward's intercept.

(2) $$\begin{array}{c} \beta_{0j}=\gamma_{00}+\gamma_{01}*\text{collective moral sensitivity}+\gamma_{02}*\text{collective general self}‐\text{efficacy}+u_{0j}. \end{array}$$

The collection of ward level intercepts, *β*_0*j*_, is modeled at Level 2 through the grand mean intercept in the population, *γ*_00_, where *u*_0*j*_ is the random effect or the deviation of ward j's intercept from the grand intercept. At Level 2, the between-ward variability is represented as Var (*u*_0*j*_).

Based on this formula, with a Type I error of 0.05 and a power of 80%, the present study required approximately 245 participants. Due to the hierarchical nature of the data, we used a power analysis that followed guidelines set out for such data.

### 3.7. Ethical Considerations

This study was conducted with the approval of the Student Research Committee of Mazandaran University of Medical Sciences and the Ethics Committee (IR. MAZUMS.REC.1402.18651). Prior to participation, all nurses were provided with detailed information about the study's purpose, the procedures involved, and the potential risks and benefits. Informed consent was then obtained from each participant, confirming their voluntary agreement to participate. Participants were assured that their responses would be kept confidential and used solely for research purposes. They were also informed of their right to withdraw from the study at any time without consequence. All data were anonymized to protect the privacy of the respondents.

## 4. Findings

Table 1 presents the descriptive statistics for the sample. Continuous variables, including nurses' perceptions of PCC, moral sensitivity, and self-efficacy, are summarized using means and standard deviations. Categorical variables, such as sex, employment status, education level, marital status, salary, and work experience, are presented as frequencies and percentages.

The results indicated that nurses' general self-efficacy was not significantly correlated with PCC (*r* = −0.010, *p* = 0.878). However, a Pearson correlation analysis revealed a statistically significant, positive association between moral sensitivity and PCC (*r* = 0.204, *p* = 0.001) (Table 2).

Moral sensitivity and general self-efficacy were significantly, positively correlated (*r* = 0.501, *p* = 0.001), which suggests that moral sensitivity and general self-efficacy increase together (Table 3).

We tested the normality of the continuous variables before conducting parametric analyses. The Shapiro–Wilk test and inspection of histogram plots were used for this purpose. Our findings suggest that moral sensitivity approached borderline normality (*p* value slightly below 0.05), while general self-efficacy and PCC significantly deviated from normality (*p* value < 0.001). However, skewness and kurtosis values for these variables (moral sensitivity: skewness = −0.14, kurtosis = 2.79; general self-efficacy: skewness = −0.52, kurtosis = 2.79; PCC: skewness = −0.83, kurtosis = 4.81) indicated moderate deviations from normality, which are often acceptable in large samples for parametric analyses. Given the sample size (*n* = 247) and the robustness of hierarchical linear modeling techniques to moderate violations of normality assumptions, we proceeded with parametric tests including Pearson correlations and HGLM.

The reported coefficients presented in Table 4 indicate the expected change in the dependent variable (i.e., person-centered nursing) for each unit change in a specific independent variable (i.e., moral sensitivity, general self-efficacy) at each level. These unstandardized coefficients indicate the direction, magnitude, and significance of the main predictors. Positive values reflect a positive relationship between person-centered nursing and the predictors, while negative values indicate a negative relationship. The larger the value, the stronger the relationship at each level. As shown, nurses' perceptions of moral sensitivity (Level 1) demonstrate a significant positive relationship with person-centered nursing (*B* = 0.22, *p* < 0.001), which suggest that moral sensitivity is a potential factor in the success of person-centered nursing.

Intraclass correlations (ICCs) were also calculated for all four models. Model 1 revealed that 13.21% of the variance in PCC was explained at the ward level. In Models 2 and 3, by adding individual variables, including control variables and main predictors, the ICC increased. However, in Model 4, after introducing contextual variables at the department level, the ICC decreased, and some of the variance was explained by these variables (collective moral sensitivity and general self-efficacy), which implies that the ward environment may reduce PCC, but high moral sensitivity may play a crucial role in PCC.

## 5. Discussion

This study examined the relationship between moral sensitivity and general self-efficacy with PCC in nurses working in the ward at individual and departmental levels. The findings indicated a statistically significant positive relationship between PCC and moral sensitivity, aligning with the results of Park et al. (2018). This suggests that nurses with higher moral sensitivity are more likely to provide PCC. However, the fact that the nurse's work environment had a more substantial effect compared to moral sensitivity suggests that the organizational factors are also important, indicating that improving the nursing work environment is crucial [12]. Also, Raeissi et al. mentioned that Iranian nurses are working under hectic work schedules with poor staffing and inadequate facilities in an unsupportive work environments which lead to high stress in them. These demanding conditions such as limited beds, equipment shortages and extended may explain the differences in outcomes like PCC [39]. As Jang et al. suggest, fostering moral sensitivity develops ethical attitudes and responses in nurses, facilitating the provision of ethical care to patients [40]. Furthermore, moral sensitivity enhances individuals' spirituality, potentially elevating the quality of caring behavior in nurses [41–43]. These findings highlight the importance of cultivating moral sensitivity among nurses to enhance PCC. This suggests that moral sensitivity is an important factor for nurses to have when using PCC.

The findings revealed no significant relationship between PCC and self-efficacy, which contrasts with some previous studies. Similarly, a study conducted in 2020 by Walling BM reported that self-efficacy did not impact care skills [44]. This finding does not align with Bandura's social cognitive theory, which posits that self-efficacy is a key determinant of behavior [15]. However, the results of a study conducted in 2021 by Hany A showed that family-based care knowledge enhances patient care by influencing nurses' self-efficacy. These varying results may be attributed to cultural differences, gender, educational status, and other conditions, making such discrepancies understandable [45]. Additionally, a study conducted by Drama et al. in 2020 identified a positive relationship between self-efficacy and caring behaviors toward patients [46], while a study in 2019 noted that nurses' initiation of caring behavior is linked to their self-efficacy [47]. This suggests that self-efficacy may not be related to any specific factor, and must have other circumstances to be of importance. Therefore, further research is necessary to clarify the relationship between these two variables.

The modeling results indicated that PCC was not influenced by moral sensitivity or self-efficacy at the department level (i.e., collective self-efficacy and collective moral sensitivity within departments). This finding suggests that individual characteristics are important factors. These findings suggest that the environment may affect personality. However, research conducted by Woods et al. in 2019 suggested that while individual personality traits are often considered stable over time, they can still be influenced and altered in adulthood by the work environment, which is a significant part of an individual's life. The work environment, which shapes daily values, social roles, and activities, can impact personality formation more than factors like marriage [48]. The environment can affect the people around it and their personality. Therefore, it may be understandable that nurses adapt to the characteristics of their specific department over time, and just as the desired variables differ among individuals, the same variables may vary across different departments.

The findings revealed a significant positive relationship between moral sensitivity and general self-efficacy, which has been consistent with other research showing a significant relationship between general self-efficacy and the moral environment [49, 50]. These results may be explained by the role of self-efficacy as a mediating variable between the perception of the moral organizational climate and the level of resistance to unethical organizational orders [49]. As a result, individuals with higher self-efficacy are likely to exhibit greater moral sensitivity. Because of the organizational climate, and high self-efficacy, the individuals are able to exert better moral sensitivity.

The regression modeling results indicated a positive relationship between PCC in nurses and their work experience, with nurses having less than 2 years of experience providing higher levels of PCC. However, studies have shown that work experience does not necessarily impact PCC [51, 52]. In contrast, a study conducted by Tiainen et al. in 2021 found that individuals with 5–9 years of experience tend to provide higher PCC [53]. Since much of nursing involves practical work that requires accumulated clinical experience, more experienced nurses are likely to provide better patient care [54, 55]. On the other hand, some studies suggest that as nurses gain more experience in a department, they develop a routine that can hinder the delivery of higher-quality care [56, 57]. Additionally, nurses with more experience may sometimes bully less experienced colleagues, such as new staff, which can negatively affect the overall quality of patient care [58, 59].

The regression model results revealed a significant relationship between PCC and the income level of nurses. In this context, a study in 2021 found that job satisfaction in nurses, which can be influenced by income and benefits, has a significant positive relationship with the level of their caregiving behaviors [60]. Also, a study indicated that Iranian nurses often perceive their salary as inadequate and unfair, which reduces job satisfaction [39]. In addition, type of contract among Iranian nurses is a crucial key in Iran which makes higher dissatisfaction and turnover report among nurses with insecure contracts [61]. However, while salaries and benefits are important factors for job satisfaction among nurses, their work environment takes precedence in practice. In this case, providing suitable working conditions could enhance nurse performance [62]. Providing a good working environment is more important to performance than salary. Conversely, another study found that job satisfaction, influenced by income, does not impact the type or quality of care nurses provide [63].

## 6. Conclusion

The level of PCC among nurses was found to be below the desired standard. With the growing elderly population in Iran and the rise in chronic diseases, implementing strategies to enhance nurses' understanding of this concept is crucial. Future initiatives should focus on moral sensitivity training, given its demonstrated ability to improve care [64, 65]. Moral sensitivity may play a key role in providing PCC; therefore, employing nurses with the necessary ethical competencies is recommended.

The results also indicated that higher self-efficacy does not necessarily translate to better PCC. Despite the high self-efficacy scores among the nurses studied, no significant relationship was found between self-efficacy alone and the delivery of PCC. Consequently, ward-related variables should be considered. These ward-level variables are influential and, when combined with self-efficacy, can create opportunities to enhance PCC, especially with the support of health officials. However, the study's limitations must be addressed in future studies, and should be accounted for.

The results also indicated that nurses with less work experience tend to provide better PCC; nursing managers may prioritize a younger workforce in hospital wards or centers where PCC is critical.

Consequently, health system policymakers should address the barriers nurses face and ensure the training of nurses capable of delivering comprehensive and targeted care for improved PCC. These findings should be considered in future research, as the study has been found to have limitations.

## 7. Limitations

One of the limitations of this study is the use of a self-report questionnaire to assess demographic variables. Also, the responses are restricted to opinions, which will not assist in getting factual results, and because participants were aware of this, there is a chance of bias. This is also because of the restricted sample size, which was limited to selected hospitals in northern Iran. The geographic restriction limits the generalizability of the data to other areas, since the geographic can differ. To improve the reliability of the results, it is recommended that future studies include a larger sample size and be conducted across multiple provinces. Since the concept of PCC can be influenced by cultural factors, conducting qualitative studies in this area is also recommended. Because of other factors such as socioeconomic factors, other studies can be beneficial in emphasizing PCC.

## Figures and Tables

**Table 1:** The mean and standard deviation of demographic variables and the level of moral sensitivity and self-efficacy and person-centered care in nurses.

**Demographic characteristics**	**Group**	***N* (%)**

Age (mean ± standard deviation)		33.60 ± 7.70

Overtime hours		64.78 ± 1.92

Sex (female, male)	Female	203 (82.2)
	Male	44 (17.8)

Marital status (single and other cases., married)	Married	151 (61.10%)
	Single and other cases	96 (38.39)

Monthly income (6–12 million toman, more than 12 million toman)	−12 million toman	146 (59.10)
	More than 12 million toman	101 (40.90)

Work experience outside the current department (years)	< 2	83 (34.40)
	2–5	47 (19)
	> 5	115 (46.60)

Employment status	Contractual	16 (6.5)
	A 5-year contractual	25 (10.1)
	Permanent	139 (56.3)
	A 2-year contractual	67 (27.1)

Education level	Bachelor	237 (96)
	Master	10 (4)

**The outcome variable**		**Mean ± standard deviation**

Person-centered care		40.80 ± 6.10

*Predictor variables*		
Moral sensitivity		85.2 ± 6.9
General self-efficacy		65 ± 7.8

*Contextual-level (wards)*		
Collective moral sensitivity		85.2 ± 1.7
Collective general self-efficacy		65.7 ± 1.8

**Table 2:** The relationship between the moral sensitivity and GSE with PCC among study participants.

Variable	PCC	
	Correlation coefficient (*r*)^∗^	*p* value
Moral sensitivity	0.204	0.001
GSE	−0.010	0.878

^∗^Pearson correlation.

**Table 3:** The relationship between the GSE with moral sensitivity among participants.

Variable	Moral sensitivity	
	Correlation coefficient (*r*)^∗^	*p* value
GSE	**0.501**	**< 0.001**

*Note:* The bold values indicate general self-efficacy.

^∗^Pearson correlation.

**Table 4:** Associations of moral sensitivity and general self-efficacy with person-centered in multilevel contextual effects modeling.

	Model 1		Model 2		Model 3		Model 4	
	Β (SE)	*p* value	Β (SE)	*p* value	Β (SE)	*p* value	Β (SE)	*p* value
*Individual level*								
Intercept	41.62 (0.75)	< 0.001	47.94 (2.43)	< 0.001	34.47 (4.94)	< 0.001	34.52 (4.92)	< 0.001
Age			−0.21 (0.08)	0.007	−0.19 (0.08)	0.011	−0.256 (0.161)	0.112
Sex (ref: female)			0.12 (0.97)	0.901	0.29 (0.95)	0.759	0.019 (0.043)	0.657
Marital status (ref: married)			−1.48 (0.85)	0.082	−1.57 (0.87)	0.056	−0.131 (0.075)	0.083
Salary (ref: 60,000,000–120,000,000 rials)			−1.18 (0.88)	0.181	−1.41 (0.86)	0.101	−0.125 (0.028)	< 0.0001

*Out of ward (ref: > 5 years)*								
< 2 years			2.34 (1.11)	0.034	2.35 (1.07)	0.028	−0.237 (0.087)	0.006
2–5 years			2.91 (1.21)	0.017	2.91 (1.18)	0.014	−0.036 (0.069)	0.599
Moral sensitivity					0.22 (0.06)	< 0.001	0.271 (0.33)	< 0.0001
General self-efficacy					−0.10 (0.05)	0.081	−0.132 (0.068)	0.052

*Contextual-level (wards)*								
Moral sensitivity							0.074 (0.446)	0.868
General self-efficacy							0.339 (0.22)	0.123
Ward-level variance	4.92 (2.88)		5.44 (3.08)		5.04 (2.93)		4.79 (13.63)	0.725
Residual variances	32.31 (2.98)		29.77 (2.75)		28.14 (2.60)		0.863 (0.167)	< 0.0001
ICC (%)	13.21%		18.27%		15.18%		4.84%	

## Data Availability

The datasets without confidential information used and/or analyzed during the current study are available from the corresponding author upon reasonable request.
